# Reminiscences of the Insane

**Published:** 1887-01-08

**Authors:** 


### Reminiscences of the Insane.

By a Mental Physician.

(*Continued from page 204.*)

Practical Use of "The Hospital."

Reference has been already made to the poetry which sometimes emanates from the hospital, in which the weary or morbid brain rests for a longer or shorter period in order to recover its full power. A short poem, of no small merit, has already appeared in the columns of this Journal, on the "Flowers of November" in regard to which we are able to add a pleasant sequel, which not only illustrates the action of a kindly donor, and the grateful feeling of the recipient, but shows the practical advantage of a periodical devoted to the welfare of hospitals and their inmates. A lady whose attention was attracted to the lines in question, sent a *cadeau* of beautiful flowers to the poet-patient from a considerable distance. The gift was warmly appreciated, and led to the composition of the following impromptu lines:—

"Dear lady fair! a gift of flowers
(To one who suffers in his chest
In hospital—with weary hours—)
Demands the warmest thanks exprest
And your sweet *cadeau's* elegance
Delighted me—and will. A glance
Is quite enough to show the taste
And hearty kindness, though unknown,
Of face of her—(no diamond-paste
But jewel pure, as this has shown)
Who knew how to encourage him
That wrote a verse or two on flowers
And showed her sympathy, where dim
The sky with fog of London, lowers.
Madam, I make my humble bow,
And kiss the bouquet ('tisn't thou!)
Excuse the freedom and the fun—!
If near you—I should *blush*, and run."

Handwriting of the Insane.

Not the composition only of patients—their letters and self-dissection—but their mode of writing is frequently highly characteristic. A medical man familiar with the insane might readily detect the source of writing shown to him, although he might find it very difficult to give his reasons in a court of law. For instance, some patients are very fond of scribbling diagonally across the paper on which they write a letter, but were this given in evidence a barrister would immediately inquire, with the necessary professional air of surprise, whether writing a letter from angle to angle is a proof of insanity, and, if so, of what form? It might be difficult to gratify this very legitimate curiosity, but none the less would a mental expert be justified in recognising the form of the epistle as one of the grounds upon which he arrived at the conclusion that the writer was of unsound mind. Some patients seem to enjoy covering the envelope with the same diagonal writing, but if the patient is in an asylum, a sceptical lawyer would no doubt suggest that the patient was poorly supplied with paper! A similar explanation would as certainly be given of a letter scarcely decipherable on account of its being so copiously crossed, and he would be a bold witness, indeed, who ventured to say that a young lady was insane because she crossed her letter; still such crossing is very common with a certain class of insane persons.

Writing in Delusional Insanity.

Mysterious lines and figures, and sometimes circles within circles, appear in the letters of patients, and may indicate unexpressed delusions, or they may represent wild and fantastic plans or inventions for the salvation of the world, or modes of locomotion which, one has to admit, enter the domain of the impossible, even in the present age of progress and invention. A barrister might retort that some of the early pioneers in the application of steam to locomotion were regarded as mad. Indeed, it is generally believed that a Frenchman was confined in the Bicêtre for alleging his discovery of the marvellous powers of steam. And although the story was a clever fiction, it is not at all improbable that similar mistakes have occurred, and that a real inventive genius has had the misfortune to be shut up in an asylum. But this is no reason for refusing to regard what, according to the lights of the present day, appears to be a wildly extravagant scheme, as an important indication, among others, of a man's having lost his reason.

Writing of Acute and Chronic Maniacs.

Everyone would expect the writing of a person in a state of active mania to be rapid and almost or altogether illegible; and so it is, as a rule, in the rare instances in which you can induce a patient to put pen to paper. We have known a case however, in which not only was the writing legible, but the composition was a rhyme of considerable merit. Then there is the writing of chronic patients, who seem to revel in the most minute accuracy the words and letters they form. Page after page of of foolscap will be sometimes carefully filled with this caligraphy. Here, again, minute accuracy in the handwriting cannot be seriously brought forward as a proof of disordered mind, yet an experienced mental physician would at a glance recognise, as highly characteristic folios of elaborate composition of this kind now lying before us. Composition in these cases may, or may not be incoherent. A few words from a composition of the latter description will suffice to show what we mean: "In Notice for the High Court of Parliament during their Session, unto her and his service of the both sex and servants; so that is appointed at the Lunatick's; and house's, and are, on chief, head, place's; figured members all in their own office, unto the Form, and especially unto them or these that are of the household or keeper's thereof, of faith,"—and so on, and so on.

Writing of General Paralytics.

The most signal change from the patient's healthy to his insane handwriting occurs in that form of mental disorder termed general paralysis of the insane, but in which those unacquainted with the symptoms would most likely fail to detect what they would understand as paralysis. And yet the formation of the words and letters would be found in the examination to stamp the case as one of the kind now referred to. Thus in a letter written by a person of fair education the writer makes such mistakes as "made" for "maid," "wether" for "weather," "intelligece" for "intelligence," etc. Letters are disjoined, and entire words omitted. "So are mine," said a friend, on our showing him a similar epistle. The only reply to such criticism is that a defective letter must be compared with the same writer's letters, written when in health. In this disorder the memory of correct spelling seems to entirely vanish or become imperfect, so that the patient endeavours to give manual expression to words in accordance with their sound; their writing becoming thus at times phonetic. The shaky writing which marks this letter cannot be realised without seeing the original or a facsimile. When the last stage is reached, the unfortunate patient may be able to make only a few lines or pot-hooks, and he ends his attempt at writing as he began when a child.

Writing of Idiots.

Of the writing of idiots and imbeciles we have not thought it necessary to speak. Obviously they are unable to write in consequence of mental defect, and laborious tuition can alone enable them to produce any legible writing at all. It is among left-handed imbeciles that *Spiegelschrift*, or mirror-writing, has been specially observed, but it is by no means confined to the weak-minded. Words are so written that, being from right to left, they present the appearance that ordinary writing would do, if placed in front of a mirror. On the other hand, the abnormal handwriting can be read by a mirror or by the impression it makes on blotting-paper. It is a frequent habit with imbeciles to read some words "backwards way," as *nug* for gun, *dab* for bad; and they have been known to read, as well as copy, whole lines in this way. We have a very good specimen of mirror-writing in our possession, written by an intelligent girl. She is left-handed, but has been educated to use her right hand also.

Mirror-writing.

Merit of Teaching Idiots.

What may be done in this direction is best understood by a visit to an Institution like Earlswood, where commiseration for the pitiable objects tended and taught in that Institution is only equalled by the wonder experienced that any teachers can be found willing to labour day after day in instructing the idiotic in the art of writing. If all tuition is regarded by the majority of persons as an unenviable task, this form of it, involving as it does so much patience and laborious repetition with so comparatively small a result, can hardly be thought of or described in adequate terms of praise. Yet, happily, teachers are found who are willing to sacrifice themselves to the terrible drudgery of even this mode of gaining a livelihood. A higher motive than filthy lucre no doubt animates many such teachers.

